# Hybridization Effect on Mechanical Properties of Basalt/Kevlar/Epoxy Composite Laminates

**DOI:** 10.3390/polym14071382

**Published:** 2022-03-29

**Authors:** Ramesh Velumayil, Anand Palanivel

**Affiliations:** Department of Mechanical Engineering, Vel Tech Rangarajan Dr. Sagunthala R&D Institute of Science and Technology, Chennai 600062, TN, India; rameshv@veltech.edu.in

**Keywords:** hybrid laminate, basalt, kevlar, mechanical analysis, morphological analysis

## Abstract

The present work investigates the fabrication of Kevlar/epoxy and basalt/epoxy and Kevlar/basalt/epoxy hybrid composite laminates and compares their mechanical properties. Mechanical characterization tests, including tension, flexural, impact and hardness tests, as per ASTM standards, were conducted on coupons cut out from the fabricated composite panels. A hand layup fabrication technique was used to fabricate composite panels with seven layers in them. Eight such laminates, with two containing pure Kevlar/epoxy and basalt/epoxy and the remaining ones containing Kevlar/basalt, were stacked in different sequences and impregnated in an epoxy matrix to provide a hybrid configuration. The microscopic examination of the fabricated laminates revealed that there was good bonding between the reinforcements and matrix material. Out of the eight composite panels including the hybrids, the ones with the pure basalt/epoxy exhibited more tensile and flexural strength than its Kevlar/epoxy counterpart due to its higher density value. The tensile and flexural strength of the hybrid laminates (i.e., combinations of basalt/Kevlar/epoxy) showed values in between pure basalt/epoxy and Kevlar/epoxy laminates in general. A similar trend was observed in terms of hardness and impact strength for the fabricated composite laminates.

## 1. Introduction

Composite materials offer better specific properties when compared to conventional metallic materials and that is the reason attributed to the widespread increased in their use in many engineering applications such as wind energy, automotive and consumer appliances [1]. In particular, in the automotive industry, material substitution efforts using advanced composite materials resulted in light weight structures that satisfied not only government and private regulatory norms but also reduced the carbon footprint to the impact on the environment without compromising functional benefits [2]. Advanced composite materials are considered as a potential replacement in the primary load carrying members, as there are many trade-offs between cost, performance, economic impact and others [3]. There have been continuous efforts among many research groups around the world to reduce the costs associated with such high-performing and advanced composite materials. Replacements of conventional composite materials were suggested in the form of natural fibers which possessed lower mechanical properties due to the chemical incompatibility between natural fibers and synthetic resins used in the matrix material [4,5]. Also, it needs to be pointed out that there is absolutely no need to consider advanced and other synthetic fiber-based composite materials in secondary load carrying members and panels which are included for a cosmetic purpose. There are plenty of natural fibers available in the market which are being used in combination with a variety of natural and synthetic resins that satisfy the need of secondary structural applications in various industrial sectors. Also, these natural fiber-based composite materials offer an overall weight reduction to the resulting structure. Among many such natural fiber-based composite materials, ones made of basalt and Kevlar play a major role in many structural applications due to their superior properties when compared to other natural fiber composites [6,7,8,9,10,11,12,13,14,15,16,17].

The properties of hybrid composite laminates obtained from mechanical characterization tests based on pure and hybrid natural fibers such as jute, bamboo and other synthetic fibers are presented in [6,7,8]. These tests showed that hybrid kenaf/Kevlar/epoxy composite laminates possess enhanced mechanical property values compared to pure laminate configurations [8]. Hybrid composite materials are a valid alternative to such conventional composite materials and offers a low density, which results in a lower weight, reduced cost due to multi-reinforcement options and enhanced properties due to the combination of reinforcements, and, more importantly, all of the above are achieved without sacrificing functionality. The mechanical and impact properties of individual and hybrid synthetic fiber-based composite materials are presented in [9,10,11,12,13,14,15,16]. The enhanced impact mechanical properties of Kevlar/flax/epoxy composite laminates in a sandwich configuration are experimentally presented in [9]. Mechanical characterization tests, such as tension, flexural and impact tests, carried out on basalt/epoxy composite laminate including different amounts of graphene Nano pellets showed that the pellets increased the material’s mechanical properties, as provided in [10]. Investigations into the mechanical properties of pure Kevlar/epoxy composite materials are presented in [11]. Kevlar is a class of aramid fibers that is used in a variety of applications. For example, it is used as a replacement for steel in racing cars and for asbestos in bicycle tires, to name a few. Recently it has found applications in the making of boats, bullet proof vests and armored plates for defense applications due to its superior specific properties compared to its metallic counterparts. It has the benefit of a low density and also exhibits desirable thermal properties such low thermal conductivity. These properties make it an excellent candidate for applications that require a high strength and heat dissipation [11]. Though there have been a variety of Kevlar fibers available in the market, one in particular, K29, is often selected a reinforcement material because of its easy availability, low cost and its extensive use in the context of cables, brake linings and defense materials.

The effects of fiber orientations and stacking sequences on the mechanical properties of glass/Kevlar, kenaf/aramid, basalt/flax, basalt/carbon and areca/kenaf hybrid composite laminates and their performances in various applications are presented in [12,13,14,15,16,17]. Basalt fibers are extracted from a naturally-occurring volcanic rock called as basalt rocks. These rock possess very fine grains which provide fibers with diameters as low as 10 to 20 µm. The fibers are then woven to obtain the required fibers. Basalt exhibits beneficial properties such as decent thermal conductivity, good elongation and low density. It also exhibits low thermal conductivity. It is used in applications such as textile fabrics, heat resistant plates and building insulation [16].

It is observed from the above literature survey that there are plenty of papers which deal with the mechanical and impact properties of natural fiber composite materials. In most of these studies, such composite laminates possess layers based on single fibers. It is observed from the literature that combining multiple fibers in composite laminates offer some advantages compared to single fiber composite laminates and some of the positives associated with this are mentioned above. Also, it is natural to understand that the sequence of such fibers in a laminate influences its mechanical properties, and the same is also reported in above literature survey. Based on the above observations from the literature survey, it is revealed that there has been limited research on the mechanical characterization of thermoset-based hybrid composite laminates fabricated using Kevlar/basalt/epoxy constituent materials. So, the objective of this paper was to determine the mechanical properties, such as the tensile, flexural and impact strength as well as the hardness, of pure Kevlar/epoxy, basalt/epoxy and hybrid Kevlar/basalt/epoxy and to present a detailed comparison of these properties. The effect of the stacking sequence and fiber volume fraction on the mechanical properties of such hybrid composite materials is also presented in this research paper.

## 2. Materials and Methods

### 2.1. Reinforcement and Matrix Material

**Figure 1:**
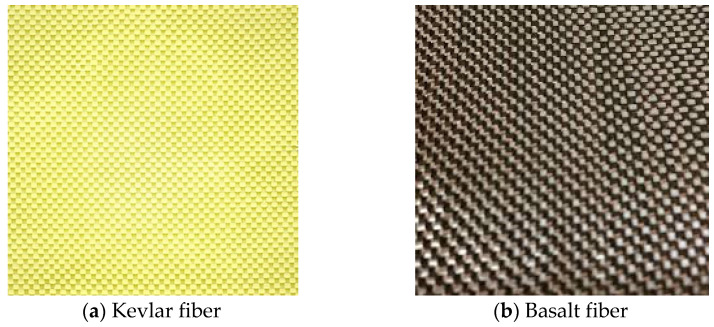
Reinforcement fiber mats, both of 300 gsm weight, used for the fabrication of hybrid composite laminates.

The present study made use of 300 gsm of Kevlar and basalt fiber mat as reinforcements, as shown in Figure 1. Also, the basic mechanical properties of Kevlar and basalt fibers are provided in Table 1. The reinforcements were purchased from Go Green Products, Chennai, TN, India.

The matrix material used for making the present pure and hybrid composite laminates are based on epoxy resin and hardener, such as LY556 and HY951, respectively. The two materials were procured from Javanthee enterprises, Chennai, TN, India. The epoxy resin used in this study is a bifunctional resin and the hardener is an aliphatic primary amine. The epoxy is typically premixed and homogenized with the hardener. The epoxy and the hardener were mixed in 10:1 weight ratio.

### 2.2. Fabrication of Composite Panels/Laminates

Two pure basalt/epoxy and Kevlar/epoxy and six different hybrid composites (i.e., basalt/Kevlar/epoxy in different stacking combinations) were produced for this study. The Kevlar and basalt fibers used in the present study are woven in nature. The matrix material was prepared by mixing the epoxy resin and its respective hardener in the weight percentage mentioned above. The reinforcements and matrix material were added in 1.5:1 weight ratios while fabricating different composite laminate configurations. The weight ratios used in fabricating the composite laminates, such as basalt/epoxy and Kevlar/epoxy, respectively, are shown in rows 1 and 2 and six different combinations of basalt/Kevlar/epoxy are shown in row 3, as shown in Table 2. In general, the composite laminates were produced by combining seven layers in different configurations. When it comes to hybrid laminate configurations in particular, reinforcement fibers are stacked in varying sequences. The hand layup technique was chosen to make the composite laminates.

The production of each hybrid composite was initiated by placing a 30 cm × 30 cm frame over a flat surface, followed by placing a waxed thin mylar sheet over the frame. The first layer of reinforcement fiber was placed on the mylar sheet. The epoxy resin mixed with the hardener was laid over the exposed surface of the reinforcement fiber and distributed evenly using a metal flat spatula. The second layer was placed over the resin, followed by a rolling process. Care was taken to ensure that the fibers were oriented with the fibers of the previous layers. The rollers were applied with even an pressure to ensure that the resin was pressed and distributed within the fibers.

**Figure 2:**
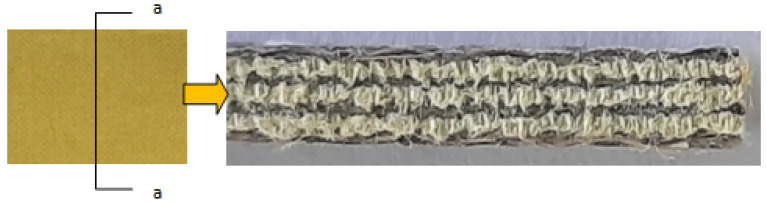
Hybrid composite laminate with a cut section showing the hybridization. Here, a-a is the cross sectional plane.

**Figure 3:**
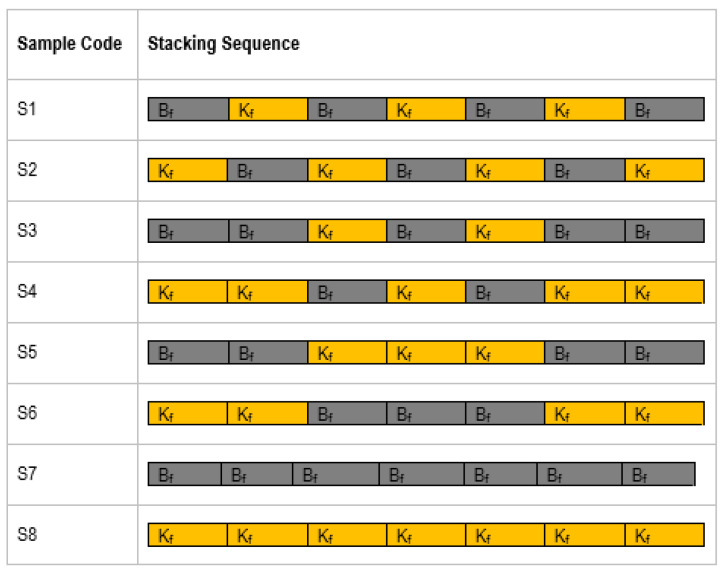
Stacking sequence of the hybrid composites.

The process was repeated until all of the seven layers of the reinforcement fibers were placed one over the other. Another mylar sheet was placed over the top layer of the composite. A uniform pressure was applied with the help of concentrated weights placed over the top surface, and the wet laminate was made to cure at atmospheric temperature for an about 24 h. The hybrid composite laminate with a cut section a-a showing the hybridization is presented in Figure 2. The six hybrid composites with different stacking sequences were produced using the same method. Figure 3 shows the stacking sequences selected for the study. Such a naming is assigned to enable the easy identification of the stacking sequences. All of the six hybrid composites were symmetrical with respect to the middle layer of the stacked reinforcements.

The total fiber volume fraction of the hybrid composites used for this study, in addition to the contribution of each fiber volume fraction to the total fiber volume fraction, are presented in Table 3. The formula used for calculating the fiber volume fraction is provided below in Equation (1) [18]. The densities of the reinforcing fibers used in the present study for calculating the fiber volume fraction are provided in Table 1.

V_f_ = (W_b_/ρ_b_) + (W_k_/ρ_k_)/((W_b_/ρ_b_) + (W_k_/ρ_k_) + (W_m_/ρ_m_)) (1)

where,

W_b_—weight of the basalt fiber,

ρ_b_—density of the basalt fiber,

W_m_—weight of the matrix,

ρ_m_—density of the matrix,

W_k_—weight of the Kevlar fiber,

ρ_k_—density of the Kevlar fiber.

### 2.3. Mechanical Characterization Tests

**Figure 4:**
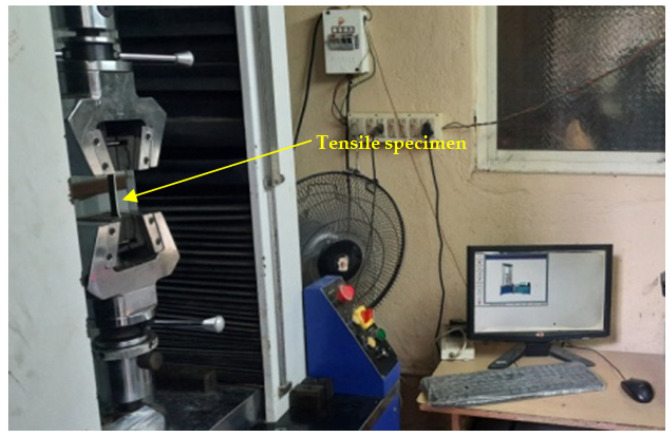
Computer controlled UTM used for tensile test.

**Figure 5:**
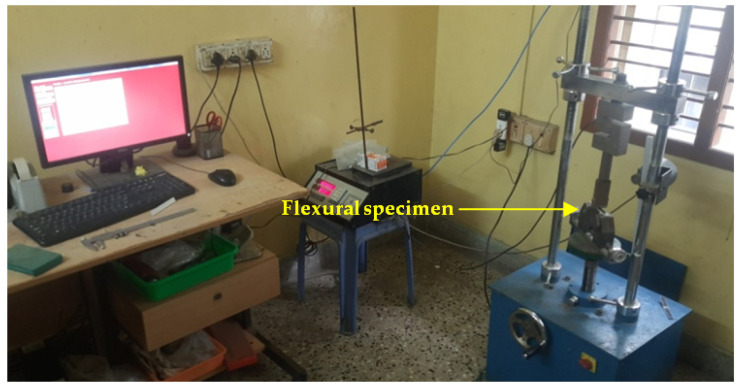
Computer controlled UTM used for flexural test.

**Figure 6:**
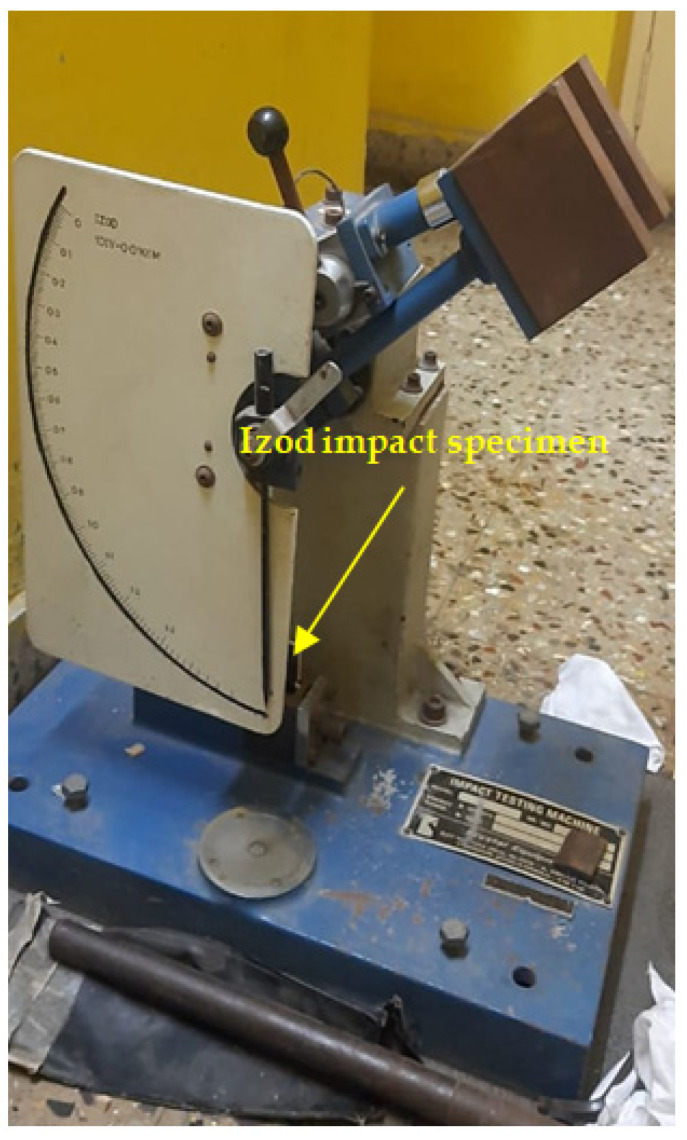
Izod impact test setup.

The fabricated composite laminates, including the six hybrids, were tested for their mechanical properties, such as their hardness and their tensile, flexural and impact strength. The coupon specimen for the tests was made as per the ASTM standards. The harness, tensile, flexural and impact tests were conducted as per ASTM D2240, D638, D790 and D256, respectively [4,5,6,7,11,12,13,14]. A UTM machine (FIE-Blue Star, Kolhapur, MH, India; Cap. 0-100kN, Model: Instron-UNITEK-94100), as shown in Figure 4, was used for the tensile tests by having a tensile grip attached to it. The same machine was used to conduct the flexural tests by changing the grip to a three-point bend set up, as shown in Figure 5. An Izod impact testing machine, as shown in Figure 6, and Shore D hardness tests equipment were used to measure the impact strength and the hardness of the fabricated composite laminates. For each mechanical characterization experiment, three samples were considered and the average of the three are reported as the mechanical property values in this paper.

**Figure 7:**
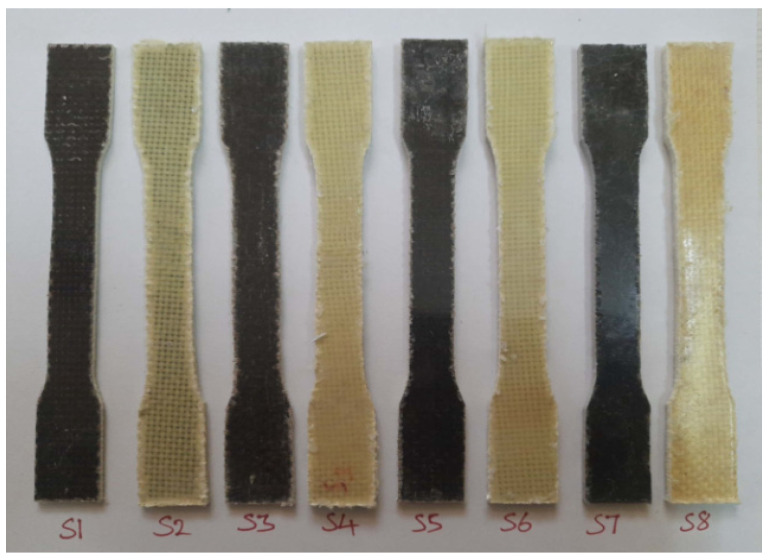
Specimens used for tensile testing.

**Figure 8:**
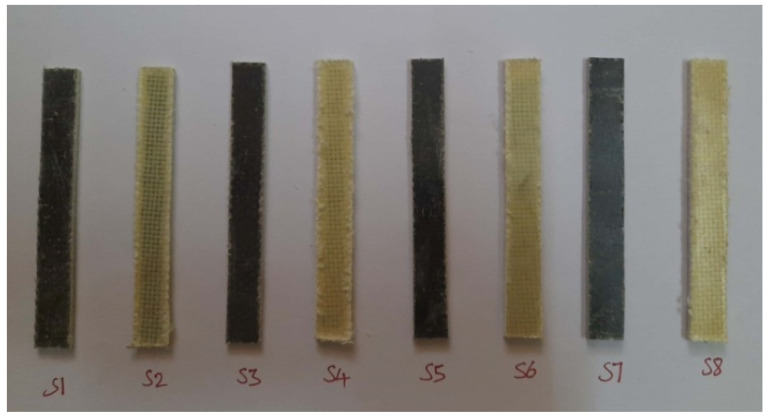
Specimens used for flexural (three-point bend) testing.

**Figure 9:**
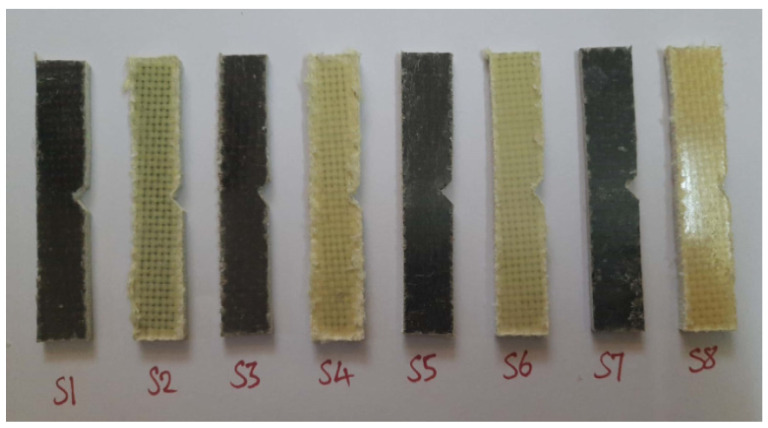
Specimens used for Izod impact testing.

The fractured surfaces of the tested specimens were analyzed using SEM (JEOL JSM 5200). The SEM analysis was carried on the fractured surface of the specimens subjected to the mechanical tests. The purpose of this was to analyze the quality of the material and also to find the nature of the failure under the load applied during the respective test. Figure 7, Figure 8 and Figure 9 show the test specimens used in this study for determining the mechanical properties. As mentioned above, the hybrid laminate configurations were coded as S1, S2, S3, S4, S5, and S6, and the remaining two composite laminate configurations for comparing the mechanical properties were coded as S7 and S8, respectively.

## 3. Results and Discussion

### 3.1. Tensile Test

**Figure 10:**
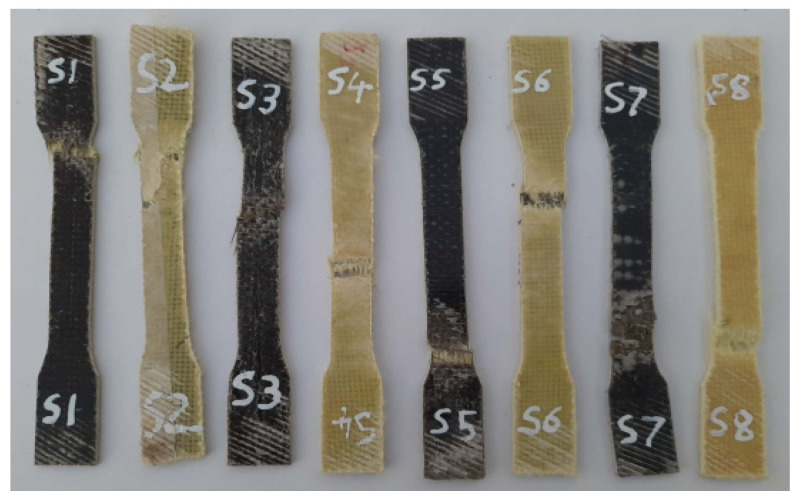
Failed specimens after the tensile test.

Figure 10 shows the images of the test specimens after the tensile tests. It can be observed that the specimens fractured between the tensile grips and at the gauge region, as shown in the below figure. Such phenomena occur under constant stress conditions arising at the gauge region during tensile testing. Also, it can be observed from the figure that in all cases, the entire specimen failed in a brittle manner and that the same can be seen with respect to stress–strain behavior as well.

**Figure 11:**
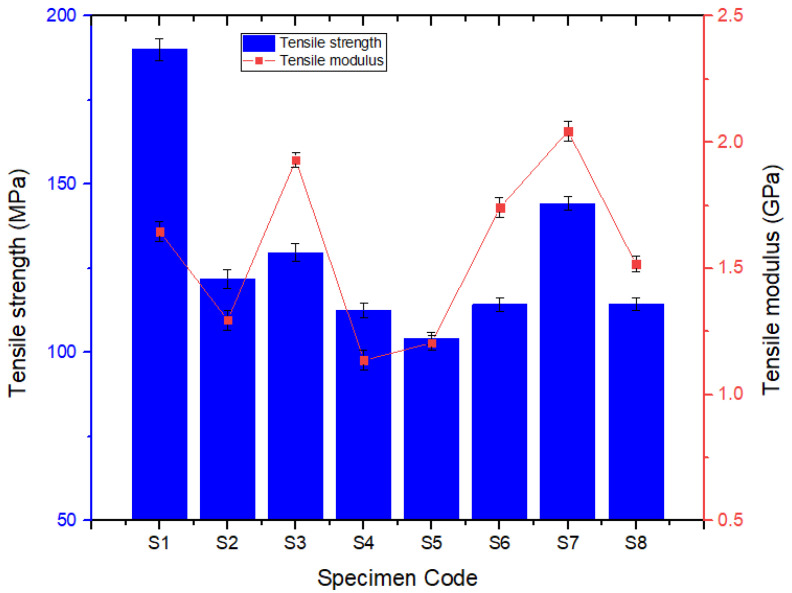
Variation in tensile strength.

Figure 11 shows the comparison of the tensile strengths obtained from the six hybrid and the two plain composite laminates used in the present study. Out of the eight specimens, the S1 hybrid composite laminate developed the highest tensile strength, 190.02 MPa, among all the laminates. Hybrid laminate S5 developed the lowest tensile strength, 104.14 MPa, among all. The remaining hybrid laminates, such as S2, S3, S4 and S6, developed tensile strengths of 121.93 MPa, 129.69 MPa, 112.55 MPa and 114.28 MPa, respectively. Similarly, pure basalt/epoxy (S7) and Kevlar/epoxy (S8) developed 144.25 MPa and 114.38 MPa, respectively, in terms of tensile strength. According to the theory behind the properties of hybrid composite laminates in comparison with pure laminates, the mechanical properties of the later fall in between the properties of pure laminates [11,12]. In this present study, the same can also be observed, except for the fact that the hybrid laminate S1 developed the maximum tensile strength. This result is considered to be an outlier and more experimental tests are required to ascertain this behavior.

The pure basalt/epoxy laminate exhibited a tensile strength that was 21% higher than the pure Kevlar/epoxy laminate, and this behavior can be attributed to the higher density of basalt fibers than Kevlar fibers. Among the hybrid laminate configurations, S3 exhibited more tensile strength than the other hybrid configurations due to the presence more layers of high-density basalt fibers. Similarly, the hybrid laminate S4 exhibited a lower tensile strength compared to the other hybrid configurations due to the presence of more layers of lower-density Kevlar fibers. Since all the laminates were fabricated using the primitive hand layup technique, it was a challenge to control the thickness of the different laminate configurations. Because, as per the theory, the laminate thickness controls the fiber volume fraction and this directly influences the mechanical properties of the fabricated laminate configurations. In terms of the tensile modulus, S3 and S7 exhibited almost equal modulus values which were higher than those of the other composite laminate configurations. This was due to the fact that S7 is a pure basalt laminate and S3 contains five layers of basalt fibers with a higher density compared to Kevlar fibers. Other laminate configurations exhibited a tensile modulus as per the presence of basalt and Kevlar fibers and their density values.

### 3.2. Flexural Test

**Figure 12:**
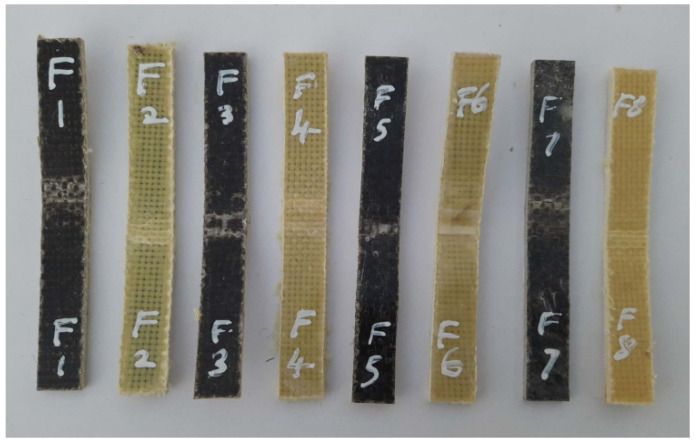
Failed specimens after the flexural test.

**Figure 13:**
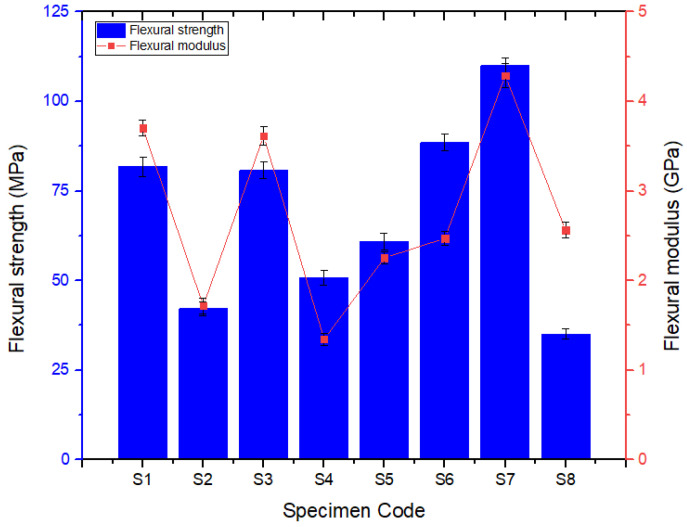
Variations in flexural strength.

Figure 12 shows the images of the test specimens after the flexural tests. It can be observed that all of the eight-specimens bent at varying proportions under the influence of the three-point bending load. This reveals that the sequence in which the reinforcement fibers were stacked in the composite materials played a vital role in the properties exhibited by the composites. However, the extent of flexural load on the specimens could not be justified from the figures. Rather, it was studied using the quantitative results obtained during the test. Figure 13 shows the flexural strengths obtained for the six hybrid composites and the two plain composites containing only basalt or Kevlar fibers. Out of the eight specimens, the one containing all seven layers of basalt fibers as the reinforcement (S7) developed the highest flexural strength, 110 MPa [10].

Its counterpart, possessing all seven layers of Kevlar fibers (S8), was able to exhibit 35.13 MPa as its flexural strength, which is 68.28% less. However, all the six hybrid composites, from S1 to S6, could only develop a lower flexural strength i.e., 8.65% to 57.68%, compared to S7. It is inferred that the layering of successive layers of the same reinforcement fibers increased the stiffness, which in turn contributed to enhancing flexural strength. Interestingly, the plain laminate S8 and hybrid laminate S2, possessing all seven layers of Kevlar fibers and alternatively stacked Kevlar fibers sandwiching the basalt fibers, respectively, resulted in diminished flexural properties. This is attributed to the ability of the matrix element to bond properly with the basalt fibers against the Kevlar fibers. The flexural modulus of the pure and hybrid composite laminate configurations used in this study exhibited a behavior which is similar to that portrayed in the flexural strength tests, as given in Figure 13.

### 3.3. Impact Test

**Figure 14:**
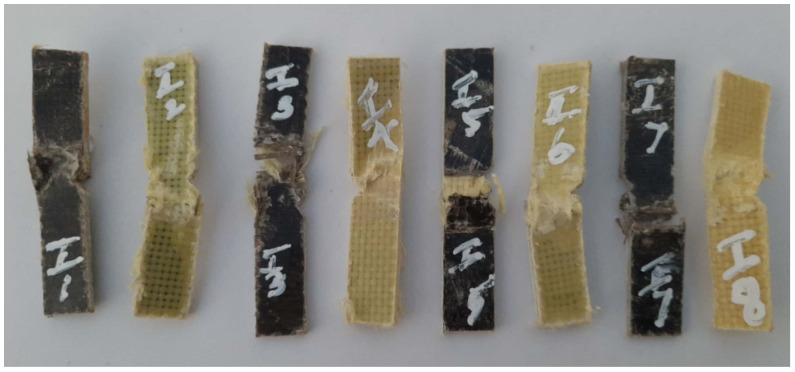
Failed specimens after the impact test.

Figure 14 shows the images of the test specimens after the impact tests. The specimen S7 fractured at locations away from the V grove in the test specimen. This reveals that the layers of basalt fibers in the composite offered resilience to the applied load. Thus, the load deviated from the point of impact. All of the other composites underwent deformation along the V groove in the respective specimen. The extent of energy absorbed was analyzed using the results obtained during the test.

**Figure 15:**
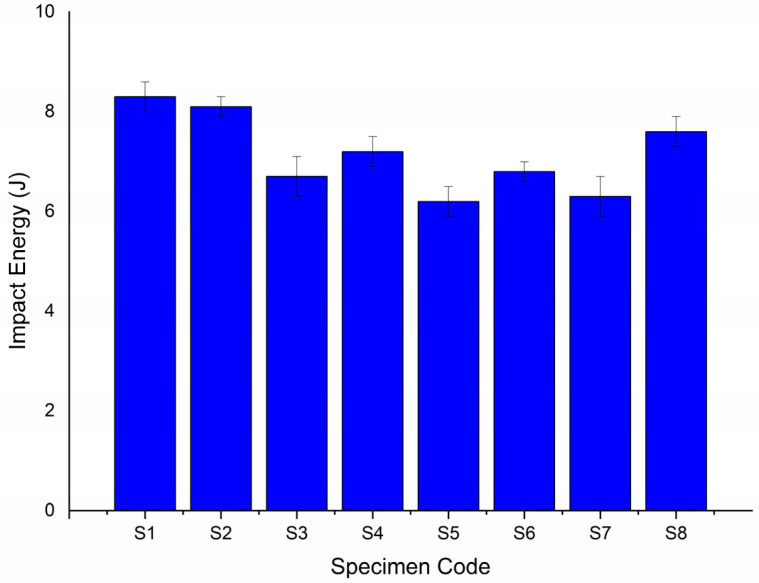
Impact energy of pure and hybrid composites.

Figure 15 shows the impact energy absorbed by the six hybrid composites and the two plain composites containing only basalt or Kevlar fibers as their composition produced for this study [14]. Regarding the impact strength of the six hybrid composites, the hybrid composite containing alternatively stacked basalt fibers sandwiching Kevlar fibers, coded as S1, absorbed the maximum impact energy, 8.3 J. Its counterparts, containing alternatively stacked Kevlar fibers sandwiching basalt fibers, coded as S2, registered a competing impact strength, absorbing 8.1 J of impact energy. However, merging two or more successive layers of basalt or Kevlar fibers, as in the case of S3, S4, S5, S6, S7, and S8, reduced the extent of impact energy absorbed by the respective composites. This is because successive layers of the same reinforcement fibers interfered in the proper distribution of the matrix element. According to the results, the later-mentioned composites absorbed less impact energy.

### 3.4. Hardness Test

**Figure 16:**
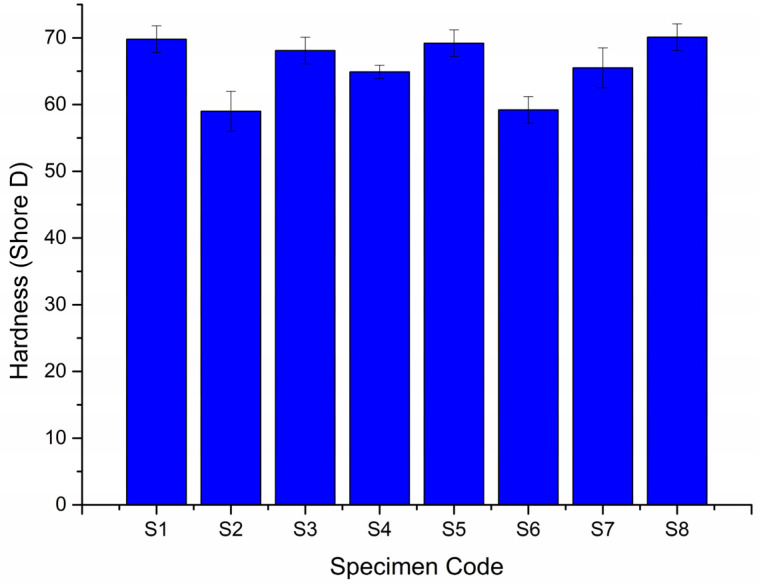
The hardness of eight composite laminates.

Figure 16 shows the hardness measured for the six hybrid and two plain composite laminates containing only basalt or Kevlar fibers as their composition fabricated for this study [8,9]. Out of the eight specimens, the one containing all seven layers of Kevlar fibers as the reinforcement (S8) developed the highest shore-D hardness, 70.1. Its counterpart, possessing all seven layers of basalt fibers (S7), was able to exhibit 6.56% less hardness. It is inferred that the Kevlar fiber was able to absorb greater hardness compared to basalt fibers. However, all the six hybrid composites, S1, S2, S3, S4, S5, and S6, could register a hardness which is comparable to S8. This shows that hybridization has an effect on the hardness of the resulting composite laminates.

### 3.5. Fractographic Analysis

**Figure 17:**
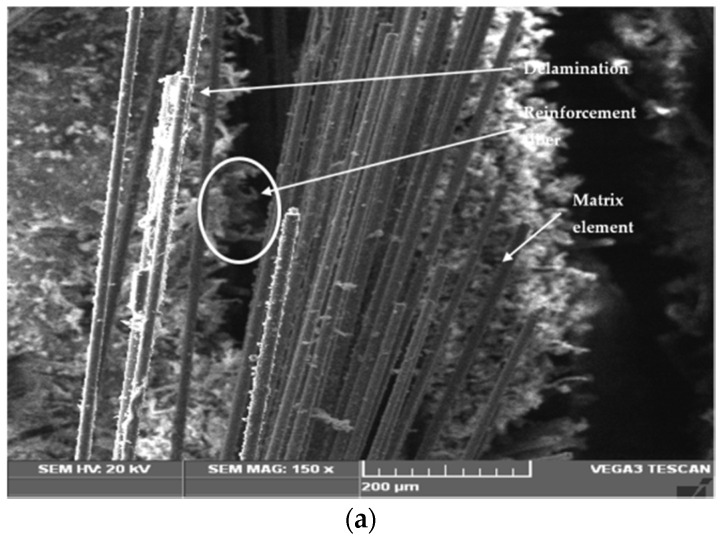

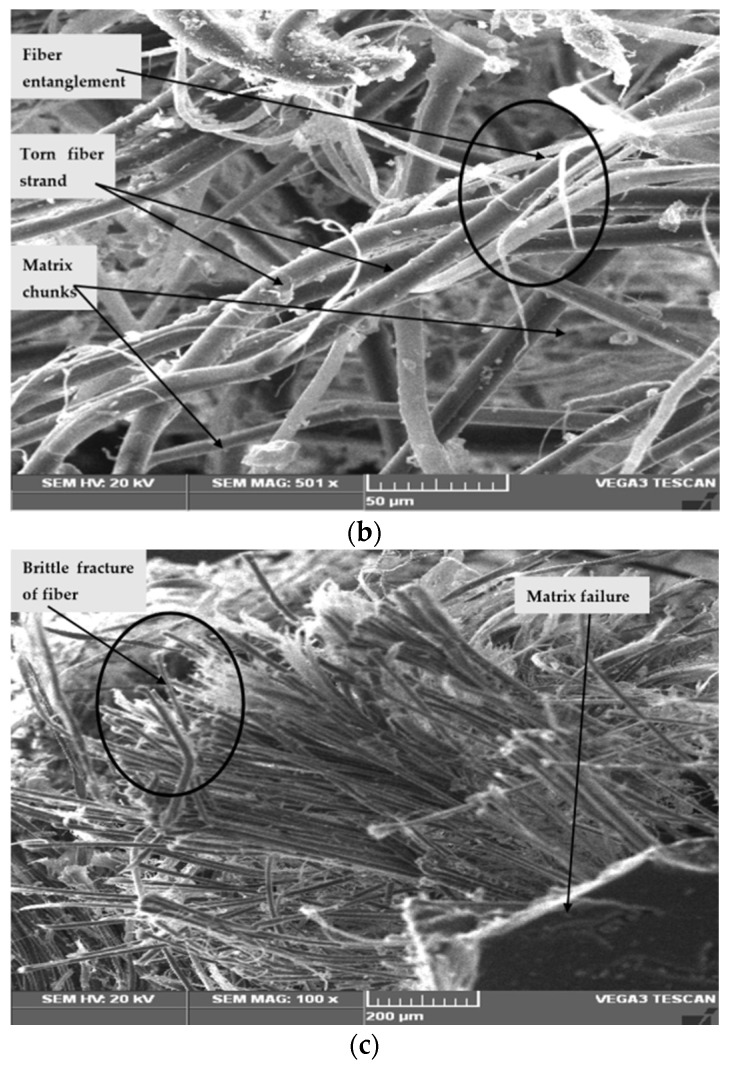
SEM of the failed specimen after (**a**) tensile test, (**b**) flexural test and (**c**) Izod impact test.

Figure 17a shows the SEM images obtained from hybrid composite S1 after the tensile test. The SEM analysis reveals that the matrix element showed good bonding with the reinforcement fibers. However, the fibers got pulled out under the influence of the tensile load. Due to the applied tensile load, the reinforcing fibers got pulled out from the matrix and fractured by snapping in a brittle manner. Also, it is inferred that during the tensile load, it is mostly the fibers that contribute to resisting the applied tensile load.

Figure 17b shows the SEM images obtained from hybrid composite S1 after the flexural test. The SEM analysis reveals that the reinforcement fibers underwent deflection due to the shear force transmitted through the three-point bending load. Because of the shear force, the matrix element crumbled and that allowed the reinforcement fibers to lose their alignment. As the result, the strands of fibers got entangled. However, a layer of reinforcement fiber just over the three-point bending load remained unaffected. This shows that the influence of the bending load affected the regions that experienced high shear strength.

Figure 17c shows the SEM images obtained from hybrid composite S1 after the impact test. The matrix element underwent a brittle mode of failure due to the impact force, as observed via the fragments of the material in the SEM image. The matrix element in all the layers in the path of the impactor crushed into smaller fragments and, according to the results, were removed. The fibers in the middle layers underwent shear deformation, and this pulled the fibers from their weave. Also, the fibers were severely damaged as the matrix element ruptured due to the impact force

## 4. Conclusions

The present study investigated mechanical characterization tests conducted on neat/pure and hybrid composite laminates fabricated using the hand layup process using basalt/Kevlar and epoxy as the constituent materials. In particular, parameters relating to the reinforcing fibers of the resulting composite laminates, including fiber volume fractions and different stacking sequences, and their effect on mechanical properties have been studied in this paper. The summary of the results obtained from the tests conducted on such composite laminates configurations are outlined below. It has been observed from the fabricated composite laminates that each hybrid and neat laminate produced a fiber volume fraction which varied from 32% up to 40%, which is in accordance with values usually associated with the hand layup process. In general, due to hybridization, the tensile, flexural and impact strengths, the modulus and hardness of the neat/pure composite laminates set the maximum and minimum values and the hybrid laminates attained values which were mostly in between those two extreme values. The above argument is true in our case, as for most of the mechanical properties mentioned above, the hybrid laminate registered respective values in between the two extreme values observed for pure/neat laminates. There are some exceptions to the above argument, in that some hybrid laminates exhibited higher mechanical properties than the maximum value attained by the pure/neat laminates, classifying them as bad performers, highlighting the need for further investigation. It is also shown from the present study that the fiber volume fraction of the fabricated laminates had a significant impact on the above-mentioned mechanical properties. SEM images taken after the experiments showed that the failure patterns observed in the present study are in accordance with the ones observed in the available literature.

## Figures and Tables

**Table 1:** Basic mechanical properties of Kevlar and basalt fibers.

Sl. No	Density (g/cc)	Tensile Strength (MPa)	Modulus of Elasticity (GPa)	Elongation (%)	Poisson’s Ratio
Kevlar	1.44	3.6	62	2.8	0.44
Basalt	2.65	4.8	110	3.1	0.2

**Table 2:** The weight percentage of reinforcement and matrix used in fabricating the composite laminates.

Sl. No		Basalt Fiber (wt%)	Kevlar Fiber (wt%)	Epoxy/Hardener (wt%)
1	1.5	0.0	1.0	
2	0.0	1.5	1.0	
3	0.75	0.75	1.0	

**Table 3:** Volume fraction of the eight composite laminates.

Sl No	Stacking Sequence and Laminate Code	Laminate Thickness (mm)	Weight (g)				Fiber Volume Fraction of Basalt Fiber b_f_ (%)	Fiber Volume Fraction, K_f_ (%)	Total Fiber Volume Fraction, Vf (%)	Matrix Volume Fraction, Vm (%)
			Weight of Composite, W_c_	Weight of Fibers, Wf		Weight of Matrix, W_m_				
				Weight of Basalt, W_b_	Weight of Kevlar, W_k_					
1	BKBKBKB (S1) (I1) (F1)	2.64	15.123	5.92	2.34	6.863	27.24	12.03	39.27	60.73
2	KBKBKBK (S2) (I2) (F2)	3.23	14.698	4.44	3.12	7.138	21.26	16.98	38.24	61.76
3	BBKBKBB (S3) (I3) (F3)	2.82	16.021	7.4	1.56	7.061	31.26	7.43	38.69	61.31
4	KKBKBKK (S4) (I4) (F4)	3.63	15.8215	2.96	3.9	8.9615	12.54	20.39	32.93	67.07
5	BBKKKBB (S5) (I5) (F5)	3.65	16.938	5.92	2.34	8.678	22.84	10.99	33.84	66.16
6	KKBBBKK (S6) (I6) (F6)	3.28	15.905	4.44	3.12	8.345	18.76	15.86	34.62	65.38
7	BBBBBBB (S7) (I7) (F7)	3.08	19.883	10.36	0	9.523	32.07	0	32.07	67.93
8	KKKKKKK (S8) (I8) (F8)	3.07	13.338	0	5.46	7.878	0	35.63	35.63	64.37

## Data Availability

The data presented in this study are available on request from the corresponding author.

## References

[1] Mallick P.K. (2007). Fiber Reinforced Composites: Materials, Design and Performance.

[2] Mallick P.K. (2010). Materials, Design and Manufacturing of Lightweight Vehicles.

[3] Elmarakbi A. (2013). Advanced Composite Materials for Automotive Applications: Structural Integrity and Crashworthiness.

[4] Asumani O.M.L., Reid R.G., Paskaramoorthy R. (2012). The effects of alkali–silane treatment on the tensile and flexural properties of short fibre non-woven kenaf reinforced polypropylene composites. Compos. Part A Appl. Sci. Manuf..

[5] Arpitha G.R., Yogesha B. (2017). An Overview on Mechanical Property Evaluation of Natural Fiber Reinforced Polymers. Mater. Today Proc..

[6] Kumar T.N.V., Mohan T.R., Sharanaprabhu C.M., Kudari S.K. (2019). Estimation of Tensile Properties for Jute Natural and its Hybrid Laminate Composites. Int. J. Eng. Res. Technol.

[7] Biswas S., Shahinur S., Hasan M., Ahsan Q. (2015). Physical, mechanical and thermal properties of jute and bamboo fiber reinforced unidirectional epoxy composites. Procedia Eng..

[8] Ramasamy M., Daniel A.A., Nithya M., Kumar S.S., Pugalenthi R. (2021). Characterization of natural-synthetic fiber reinforced epoxy based composite—Hybridization of kenaf fiber and kevlar fiber. Mater. Today Proc..

[9] Sarwar A., Mahboob Z., Zdero R., Bougherara H. (2020). Mechanical Characterization of a new kevlar/flax/epoxy hybrid composite in a sandwich structure. Polym. Test..

[10] Bulut M. (2017). Mechanical Characterization of Basalt/Epoxy composite laminates containing graphene nanopellets. Compos. Part B Eng..

[11] Rajesh S., Ramnath B.V., Elanchezhian C., Abhijith M., Riju R.D., Kathir Kishan K. (2018). Investigation of Tensile Behavior of Kevlar Composite. Mater. Today Proc..

[12] Srivathsan A., Vijayaram B., Ramesh R., Gokuldass (2017). Investigation on Mechanical Behavior of Woven Fabric Glass/Kevlar Hybrid Composite Laminates Made of Varying Fibre Inplane Orientation and Stacking Sequence. Mater. Today Proc..

[13] Yahaya R., Sapuan S.M., Jawaid M., Leman Z., Zainudin E.S. (2016). Effect of fibre orientations on the mechanical properties of kenaf–aramid hybrid composites for spall-liner application. Def. Technol..

[14] Fragassa C., Pavlovic A., Santulli C. (2018). Mechanical and impact characterisation of flax and basalt fibre vinylester composites and their hybrids. Compos. Part B Eng..

[15] Dhuban S.B., Karuppanan S., Mengal A.N., Patil S.S. (2017). Effect of fiber orientation and ply stacking sequence on buckling behaviour of basalt-carbon hybrid composite laminates. Indian J. Eng. Mater. Sci..

[16] Fiore V., Scalici T., di Bella G., Valenza A. (2015). A review on basalt fibre and its composites. Compos. Part B Eng..

[17] Sathyaseelan P., Sellamuthu P., Palanimuthu L. (2020). Influence of stacking sequence on mechanical properties of areca-kenaf fiber- reinforced polymer hybrid composite. J. Nat. Fibers.

[18] Yashas Gowda T.G., Vinod A., Madhu P., Kushvaha V., Sanjay M.R., Siengchin S. (2021). A new study on flax-basalt-carbon fiber reinforced epoxy/bioepoxy hybrid composites. Polym. Compos..

